# The relationships between students’ comprehension of conversational implicatures and their achievement in reading comprehension

**DOI:** 10.3389/fpsyg.2022.977129

**Published:** 2022-10-13

**Authors:** Safiye Çiftlikli, Özcan Demirel

**Affiliations:** ^1^Department of Modern Languages, Cyprus International University, Nicosia, Cyprus; ^2^ELT Department, Faculty of Education, Cyprus International University, Nicosia, Cyprus

**Keywords:** conversational implicatures, pragmatic competence, multiple-choice discourse completion test, reading comprehension, correlational study

## Abstract

The most important thing in effective communication is understanding not only what is said, but also why it is said. Therefore, the development of pragmatic competence in another language is essential to be able to communicate effectively. Pragmatic competence plays an important role in enabling interlocutors to work out what is intended by what is said. In this sense, special emphasis should be placed on the pragmatic aspects of language in order to enable language learners to use language appropriately. In this regard, this study aims to investigate whether there is a relationship between students’ comprehension of conversational implicatures and their achievement in reading comprehension. To this end, the data were collected from first-year 122 students at one of the private universities in northern Cyprus with different bachelor’s degrees *via* the Multiple-Choice Discourse Completion Test (MCDCT) and the reading test. The quantitative data were analysed by means of A Pearson Correlational Analysis, Simple Linear Regression, and Canonical Correlational Analysis. The results of the study revealed that comprehension of conversational implicatures of first-year university students is positively related to their achievement in reading comprehension. Moreover, it has been depicted that among the eight implicature types, topic change, indirect refusal, and disclosure are more related (0.855) to reading comprehension. Therefore, these three implicature types provide the most contribution to the participants’ comprehension of conversational implicatures. As it is, they are more powerful predictors of reading comprehension. In addition to these results, there is only one high positive correlation among the six reading subskills; that is between the subskill to identify ideas and opinions of the writer and the subskill to scan a text to find specific information (0.749). In the light of the findings, this study yields crucial implications for language teachers, material developers, and curriculum designers to take full advantage of these associations for promoting EFL learners’ achievement in reading and comprehension of conversational implicatures in the target language.

## Introduction

Effective communication in the target language is one of the most important goals of foreign language learners; therefore, developing pragmatic competence is essential to enable language learners to be able to use the target language for communicative purposes effectively. Lacking pragmatic competence, language users may not be able to understand the intended meaning properly, so the interlocutors may have difficulty in understanding what is said and why it is said. For developing foreign language learners’ pragmatic competence, comprehension of pragmatic meaning has taken a growing interest in the field of foreign language learning (Taguchi, 2005; Lee, 2018; Abdurahmonov and Kozokova, 2021; Mao and He, 2021). Understanding the meaning of an utterance and understanding the speaker’s intended meaning are two important elements of pragmatic comprehension (Thomas, 1995). The intended meaning can sometimes be different from the literal meaning of a language expression. In this sense, various studies have been conducted to develop foreign language learners’ abilities in comprehending speakers’ implicitly stated meaning (Carrell, 1984; Kasper, 1984; Takahashi and Roitblat, 1994; Garcia, 2004; Roever, 2005; Taguchi, 2005, 2007, 2011, 2013; Holtgraves, 2007; Feng et al., 2017; Derakhshan, 2019; Anggrarini and Rosdiana, 2020; Timpe-Laughlin and Youn, 2020). These studies agreed that comprehension of implied meaning is closely related to the language learners’ proficiency level in English. Development of this level in English tends to be positively correlated with the comprehension of conventional and nonconventional implicatures.

More importantly, the issue of implicatures matters a great deal to pragmatic competence. Therefore, special emphasis should be placed on implicatures to enable language learners to communicate effectively. To be able to understand implicatures clearly, it is important to understand the distinction between “what is said” and “what is implied” (Horn, 2005; Yeboah, 2021). While “what is said” semantically means the truth-value of the utterance, “what is implied” is the intended meaning of what is said by the speaker. Therefore, what the speaker implies can sometimes be totally different from what is explicitly stated. In this case, why it is said is more diverse than what is said.

There are numerous studies assessing diverse aspects of implicatures as pragmatic competence such as the relationship between pragmatic competence and language proficiency as well as the factors affecting learners’ competence to comprehend implicatures. However, there is scarcity of literature on investigating the relationship between comprehending conversational implicatures and achievement in reading comprehension. The conducted studies on the conversational implicatures agreed on the fact that comprehension of implied meaning is closely related to the language learners’ proficiency level in English (Carrell, 1984; Kasper, 1984; Takahashi and Roitblat, 1994; Garcia, 2004; Roever, 2005; Taguchi, 2005, 2007; Holtgraves, 2007; Eslami and Eslami-Rasekh, 2008; Abdelhafez, 2016). Moreover, several studies have revealed the difficulties faced by L2 learners in developing pragmatic competence, especially implicatures (Bouton, 1992; Roever, 2005; Bardovi-Harlig, 2010; Mohamed, 2022). Bouton (1992) argues that language exposure is one of the significant factors in developing the comprehension of implicatures. Roever (2005) claims that the L2 learners’ English proficiency contributes to their comprehension of conversational implicatures. In this case, his findings show that their proficiency level has a statistically significant predictive effect on comprehending conversational implicature. According to the findings of Murray (2011), L2 learners’ cultural factors can affect their comprehension of implicatures in English. Furthermore, Taguchi (2005, 2007, 2009) investigated comprehension of conversational implicature *via* a listening test. She conducted a study to look into the effects of implicature types on accuracy and speed of comprehension, the effects of language proficiency on comprehending implicature types, and the correlation between accuracy and comprehension speed. In this case, existing literature on comprehension of conversational implicatures mainly highlights the effects of L2 learners’ pragmatic competence on their proficiency level and the role of training on conversational implicatures in the development of pragmatic competence and language proficiency.

According to our knowledge, the scarcity of existing literature on the relationship between students’ comprehension of conversational implicatures and their reading comprehension, this study aims to fill this gap in the literature by investigating to what extent there is a relationship between comprehension of conversational implicatures and comprehension of written texts. Therefore, this study provides crucial insight in terms of investigating whether comprehension of conversational implicatures is one of the pivotal variables that have a relationship with the second language learners’ achievement in reading comprehension, and it also aims to clarify to what extent language learners’ achievement in reading comprehension is related to their comprehension level of conversational implicatures considering implicature types.

## Theoretical framework

### Importance of pragmatic competence in learning English as a foreign language

It is a common belief that “The more proficient you are, the better you can express yourself.” However, it is important to bear in mind that to be able to have effective communication, understanding what is said and why it is said is deeper than language proficiency. If you do not understand the implied meaning, individuals may face difficulty in maintaining effective communication in the target language. However, in the era we are living while multilingualism has taken a crucial place in almost every part of the world, English is regarded as the *lingua franca* among more than 6,500 spoken languages in the world today. The growth of scientific and technological progress has made our world a village, and this village needs a common language that is widely recognized. Having progress in science and technology has created a necessity to use English as an international language in order to have effective communication across different cultures and nations. It becomes the most widely recognized global language all around the world and is accepted as a significant tool in various fields like scientific communication, the business world, and political issues (Karatas et al., 2016; Sunitha et al., 2021). Therefore, individuals’ ability to use English for communicative purposes becomes one of their most paramount survival necessities in this globalized world. This necessity sheds light on considering how to teach English as a foreign language effectively and what factors might be related to foreign language learning. In this sense, with the rapidly increasing necessity and attention to learning English as a foreign language, it is utterly crucial to carry out in-depth analysis to understand the possible factors that are related to the process of foreign language learning. Researchers studied various issues like age, gender, nationality, learning styles, and so on to clarify what provides contributions and also what creates obstacles in the process of language learning. To provide endeavour in facilitating foreign language learning, many scholars agreed on the fact that pragmatic competence is a full-fledged part of language competence, and it becomes an indispensable issue in second or foreign language learning.

Effective use of the target language in a communicative context is an arduous process and there are various contributing factors to being able to comprehend utterances. Pragmatic and contextual information have significant roles in terms of determining what a speaker wants to convey and what a listener needs to comprehend the intended meaning (Blakemore, 1992; Sperber and Wilson, 1995). In the study of communication, context is usually apprehended as an extensive and multidimensional concept due to including cognitive and social dimensions as well as linguistic, physical, and other non-linguistic features (Sperber and Wilson, 1995). For effective communication, the hearer needs to interpret the language expressions by means of utilizing the intended meaning in a context. While the same expression can be deduced differently with respect to different contexts and an individual’s world knowledge, pragmatic competence enables language users to find a relationship between what a speaker says and what he/she actually means. In this sense, although the vast literature is usually related to the good characteristics of language learners, promoting language learners’ pragmatic competence is unquestionably a serious issue in the domain of learning English as a foreign language.

The communicative approach in foreign language teaching inaugurates a theory of language as communication. According to Hymes (1972) “communicative competence” is achieved *via* both the knowledge of a language and the ability to use it. This term has been coined by Hymes to contrast the communicative conception of language and the linguistic theory of Chomsky on competence. According to Chomsky’s claim, competence qualifies speakers to produce and understand a various number of sentences by distinguishing grammatically correct sentences from ungrammatical sentences. In Chomsky’s view, competence is an ideal language system, and it can be studied independently under “performance.” He supports his claim by emphasizing that competence is determined by the ideal speaker-hearer’s knowledge of the language and the ‘mental reality’ which is based on all aspects of language use. In contrast to the claim of Chomsky, Hymes contends that competence is what a speaker needs to know to be competent in communication. In this respect, individuals’ communicative competence is a crucial constituent in terms of determining their knowledge and ability to use language appropriately. In this sense, as opposed to what Chomsky claims, Hymes’ theory of knowing about a language provides a high-grade comprehension view.

As opposed to the model shown in the study of Canale and Swain (1980), the non-grammatical features of language ability have attracted the attention of Bachman (1990, p. 84), who categorizes language competence into two main categories: pragmatic and organizational components. This model categorizes pragmatic competence individually rather than as a subcategory of sociolinguistic competence. In this case, individuals’ pragmatic language ability has taken prominent notice in communicative competence paradigms. Whereas the ability to use language in a socially appropriate way is important, having grammatical competence should not be underestimated. Apparently, the study of Bardovi-Harlig (1996, p. 21) has predominantly focused on whether a significant correlation exists between proficiency level and pragmatic competence, and the results of the study demonstrate high grammatical proficiency does not tend to be associated with high pragmatic competence. Another interesting study that concurs with the findings of Bardovi-Harlig is the study of Jianda (2006), which reports that a learner with a high TOEFL score will not necessarily have high pragmatic competence. Although findings show that language learners are in trouble with how to develop their pragmatic competence, it is required to conduct a thorough study to develop their pragmatic competence, since inability to develop pragmatic competence may lead to serious communication problems. In Latin communication means “communicare,” which means “to share” or “to make common.” It requires understanding and sharing information in the process of interaction between individuals. If an effective relationship is established between speaker and hearer in terms of understanding and sharing information, effective communication is held. In the process of understanding, individuals are required to perceive, interpret, and find a connection between their perception and interpretation of what they know (McLean, 2003). In this sense, understanding the words and what they actually mean is an utterly crucial part of the communication process. The communication process is the precise way towards attaining effective communication. Although it seems straightforward, actually it is not. Certain barriers can provide obstacles to the communication process, and these obstacles may affect communication negatively like using an inappropriate medium, incorrect grammar, the words that conflict with body language, and specialized language relating to a particular subject or profession. Therefore, understanding the communication process will lead to being more effective communicators.

### How to develop students’ pragmatic competence

Pragmatics plays a pivotal role in having effective communication for second language speakers; therefore, it requires second language teachers to consider the ways of developing learners’ pragmatic competence. Techniques can be dissected into three categories: (1) cognitive-awareness raising activities like presentation, discussion, and pragmatic-consciousness raising techniques; (2) receptive-skill development by implementing teacher premeditated materials; and (3) productive-skills teaching accounting of role-playing (Judd, 1999). Moreover, it is claimed that although it does not ensure the development of pragmatic competence successfully, it is utterly crucial to use authentic L2 input. Therefore, language learners will be provided opportunities to discerning the language in communicative practices (Kasper, 1997; Zangoei et al., 2014). Although there are miscellaneous alternatives for authentic materials, Celce-Murcia (2001) supports the use of authentic audio-taped materials. Furthermore, consciousness-raising tasks are also strongly supported by many scholars (Judd, 1999; Barekat and Mehri, 2013; Birjandi and Derakhshan, 2014; Pourcheragh, 2019) for developing students’ pragmatic competence. They commonly accentuate that language classes in which consciousness-raising tasks are applied show better performance compared to other groups. Consequently, providing various kinds of learning opportunities is expected to facilitate foreign language learning tremendously. In this sense, integrating various activities is expected to be effective in terms of facilitating language learners’ pragmatic competencies and making ease in terms of understanding pragmatic contrasts between students’ first language and target language.

Although considering the abovementioned techniques is worthy to facilitate language learners’ pragmatic competence, measurement is also important to be able to diagnose both strengths and weaknesses. Generally, measurement is simply straightforward as it is measured concerning what is tested. For instance, accuracy and reaction times are measured with judgment tasks or selection of the correct options with multiple-choice tasks. However, measurement is difficult to determine with the task in conversation and the various conversation simulations (Bardovi-Harlig, 2013). For instance, as Aufa (2016) indicates some effective tools that are used to assess students’ pragmatic competencies are role plays, multiple-choice oriented questionnaires, rating scale assessments, simulations, interviews, and Written Discourse Completion Test (WDCT).

On the other hand, regarding its practical use, representing students’ pragmatic competencies, showing how learners use language as a tool for communication within the context compared to other assessment tools, the popularity of the WDCT has been apparently accepted (Hudson et al., 1995; Jie, 2005; Roever, 2005; Jianda, 2006; Xu and Wannaruk, 2015; Aufa, 2016; Tajeddin and Bagherkazemi, 2021; Bagherkazemi and Harati-Asl, 2022).Considering the reliability and validity aspects, the WDCT has been approved, and it can be applied as a valid and avail assessment of language learners’ pragmatic competencies. Therefore, it can be used as one of the effective alternatives for assessing students’ pragmatic competence to facilitate students’ learning target language in terms of diagnosing students’ weaknesses and strengths. As it is, it provides ease in terms of taking precautions to minimize the weaknesses of the students resulting from a lack of ability of pragmatic competencies including conversational implicatures.

### Conversational implicatures covered in the present study

Teaching second/foreign languages has experienced new trends and methods to promote communication effectively (Bozdoğan, 2015; Abrams, 2017). Communicating appropriately and successfully needs not only linguistic competence but also pragmatic knowledge (Thomas, 1995; Shokouhi and Rezaei, 2015; Choraih et al., 2016; Nourdad, 2022). According to Grice (1989), effective communication occurs if what our words say or imply corresponds to what we imply in uttering them. Although mastering grammar and vocabulary may help learners produce correct grammatical sentences, those sentences may not be appropriate in specific contexts. In this case, failure in comprehending conversational implicatures may lead to misunderstandings among interlocutors (Tsvetkova, 2022). Taking account of all mentioned above, “Conversational implicatures” were addressed as the focal point of the present study. Table 1 lists the mechanisms used for communication of implied meanings included in the study with their quantities in the data collection instrument, and then briefly explains the rationale behind their inclusion.

**Table 1:** The numbers of the test items in each group of implied meanings and their sources.

Implied meaning number of test items source		
Pope questions (X1)	5	Bouton (1994)
Indirect criticism (X2)	4	Bouton (1994); Kubota (1995)
Topic change (X3)	4	Roever (2005)
Indirect advice (X4)	4	Matsumura (2001, 2007)
Verbal irony (X5)	3	Colston and O’Brien (2000)
Indirect refusals (X6)	3	Taguchi (2005)
Disclosure (X7)	3	Taguchi (2005)
Indirect requests (X8)	2	Rinnert and Kobayashi (1999)

Firstly, it should be mentioned that some of the mechanisms used to introduce implied meanings listed above (Pope Questions, Indirect Criticism, Verbal Irony, Topic Change and Disclosure) have already been included in several other studies (Bouton, 1994, 1999; Roever, 2005). However, Indirect Advice, Indirect requests, and Indirect Refusals have not been used with the abovementioned implied meanings in any data collection instruments. Çetinavcı and Öztürk (2017) bunched them all together, and they developed “The Multiple-choice Discourse Completion Test” as a data collection instrument to assess the participants’ comprehension of conversational implicatures.

#### Pope question

This kind of question communicates an implicature using an obvious fact stated in a question. The basic form of POPE-Q implicature is as follows:

*Context: A mother and her daughter Jenny have been discussing the upcoming weekend. Jenny’s parents are leaving town and this is the first time Jenny has been left at home alone*.

*Mother:* Are you sure you can take care of yourself this weekend?

*Jenny:* Can a duck swim, Mother? (Bouton, 1988, p. 193).

Jenny answers a YES/NO question with another question. For the implicature to work, Jenny’s mother asking the first question will understand the implied meaning if she knows that the answer to her question is the same as the answer to the second one.

#### Indirect criticism

It is also called “Understated Negative Evaluation” or “Damning with Faint Praise.” It happens when we do not want to say explicitly what we think of something or someone that we, in fact, do not like. Instead, the implied meaning is conveyed *via* criticizing indirectly, like commenting about an unimportant feature of the item to imply that there is nothing else to be praised.

It is exemplified below:

*Context: Two teachers are talking about a student’s term paper.*

*Mr. Ranger:* Have you read Mark’s term paper on modern pirates?

*Mr. Ryan:* Yes. I read it last night.

*Mr. Ranger:* What did you think of Mark’s term paper?

*Mr. Ryan:* I thought it was well-typed (Bouton, 1988, p. 193).

Mr. Ryan does not provide enough information to satisfy Mr. Ranger’s request. This under-informative utterance leads Mr. Ranger to infer that Mr. Ryan did not like the essay.

#### Topic change

Topic Change is another device communicating implied meaning which happens when a person does not like what has just been said or asked, s/he leaps into another topic. See the following example:

*Context: Bob and Maggie, friends, are talking about school and courses. Bob is taking introductory chemistry this semester.*

*Maggie:* “How are you doing in chemistry?”

*Bob:* “So … did you watch that basketball game yesterday?”

As it is seen, Bob does not seem to like the current line of the conversation. Therefore, instead of satisfactorily replying to Maggie’s question, Bob presents another topic at that moment of the talk by asking an irrelevant question.

#### Indirect advice

Indirect Advice is one of the under-investigated mechanisms used for communication of implied meanings like “indirect requests” and “disclosures” in a MCDCT format. In line with Searle's (1979) classification of illocutionary acts, Advice is considered as a directive. It is defined as “telling you what is best for you” (Searle, 1969, p. 7). According to Matsumura (2001), the speaker uses the speech act of Advice like suggestion or recommendation to make the addressee act in a certain way. Therefore, the speaker’s intentions are not stated explicitly (Levinson, 1983; Brown and Levinson, 1987). The following is an example of Indirect Advice.

*Context: Judie and her classmate David are community college freshmen. Judie is considering taking a course, but David has heard it is really difficult.*

*David:* “I do not know … but people say it’s really difficult.”

As shown here, David advises indirectly not to take the course by just giving the reason why Judie should not take the course.

#### Verbal irony

Verbal irony is the use of words to convey a meaning that is especially the opposite of the literal meaning of the words. It is a type of implied meaning that implies something distinctly different, even often contrary to what is literally said. It is commonly defined as saying something indirectly while the underlying meaning is the opposite (Barbe, 1995; Sukmaningrum, 2016). In this case, verbal irony should include features that assist the addressee in comprehending correct interpretations to maximize relevance. The following is an example:

*Context: Peter promises his friend Mary to help her move to a new apartment. That day, he moves the clock on the wall while Mary moves the heavy boxes.*

*Mary:* “Thanks, you have been terribly helpful.”

Mary’s statement shows that there is a disparity between reality and utterance. Mary implies that she feels dissatisfied with Peter’s help and expresses her feeling with a sarcastic remark.

#### Indirect refusals

Utterances containing “Indirect Refusals” are viewed as a formulaic way of implying meanings. Interlocutors tend to use indirect refusals to refuse something so that more polite expressions are uttered to decrease the negative impact of refusal in various situations. Therefore, it happens when the refusal is performed *via* other verbal messages to camouflage and conceal the speaker’s true intention. For example, an interlocutor may use an indirect refusal to refuse an invitation by saying, “I have a really busy schedule this week.” Another example of Indirect refusal is provided below:

*Context: Jack sees his classmate Jane in the faculty hallway.*

*Jack:* “Oh, Jane. I’m so glad I ran into you. I need your help!”

*Jane:* “What’s up?”

*Jack:* “I have a paper due tomorrow, but I’m working tonight in the cafe. Can you type my paper?”

*Jane:* “Shoot! I have to study for my finals tonight.”

In this example, Jane shows indirect refusal using the regret refusal strategy. Jane’s statement shows that she *does not refuse to comply with Jack’s request* with explicit linguistic markers of refusals like “I cannot,” “No,” or “I do not want to,” which were exemplified as direct refusal expressions by Beebe et al. (1990; as cited in Taguchi, 2007, p. 321).

#### Disclosure

Disclosure is another type of device communicating implied meaning covered in this study. It assists interlocutors in avoiding disclosing embarrassing information (Taguchi, 2002, p. 157). For example, when an interlocutor is asked about the reality of something, and the answer makes him/her give embarrassing or disturbing information, s/he might give reason(s) about the consequence. By doing so, the interlocutor provides an indirect answer about the reality that is being questioned. See the following example:

*Context: Susan and Tom, friends, are talking about what is going on in their lives. Susan knows Tom had a job interview recently.*

*Susan:* ‘So how was your interview? Did you get the job you applied for?’

*Tom:* ‘Um … I think I need to improve my interview skills.’

As is seen, Tom does not respond to Susan’s question by using a direct answer of confession about why he could not get a job. In that way, his reply indirectly makes the revelation. He replies by giving the reason from his point of view.

#### Indirect requests

Another type of device communicating implied meaning covered in the study is Indirect Request, also called Requestive Hints (Weizman, 1985, 1989, 1993; Rinnert and Kobayashi, 1999) in the pertinent literature. *Indirect requests* are usually defined as non-conventionalized since no conventional forms are provided. They are often conceptualized as an instance of non-literal language, and their comprehension is often explained within the framework of conversational implicatures (Terkourafi, 2009; Green, 2010; Marocchini et al., 2022). According to Márquez Reiter (2000), interlocutors may perform indirect requests by using conventionally indirect requests (e.g., Would you mind giving me a hand?) or a non-conventionally indirect request (e.g., This is so heavy!; p. 42). Therefore, interrogative or declarative structures are used to make requests indirectly. For an example of the declarative structure, see the situation and dialogue below:

*Context: Nina, an office secretary at a university, is working at her desk. Tom, a teacher, is there to make photocopies but the machine is not working.*

*Tom:* ‘The copy machine is not working.’

An indirect Request is exemplified above to show how Tom wants help from Nina with fixing the copy machine.

### Importance of conversational implicatures for developing language users’ pragmatic competences

Learners of a second language can communicate effectively by interacting, negotiating meaning and having transactional communication in the target language (Celce-Murcia et al., 1995). However, it is pivotal to take into account that second language learners may have limited language input (Bardovi-Harlig, 2010; Baroto, 2017; Rinker et al., 2022). Thus, limited pragmatic input may cause failure in comprehending and using pragmatic features of the target language, including comprehension of conversational implicatures.

Conversational Implicatures as a topical issue in pragmatics has created consequential awareness in almost all pivotal modern pragmatics studies (Grice, 1975; Leech, 1983; Levinson, 1983; Brown and Levinson, 1987; Thomas, 1995; Cummings, 2005). It becomes one of the topical issues in literature in the case of yielding up the following:

- It is considered the most prevailing phenomenon in pragmatics.
- It has taken tremendous attention to creating awareness of how intentions may vary depending on different utterances.
- It can be considered a simplification of the language structure and the content of the semantic description.

It also highlights the issue that studying some aspects of a language semantically is not enough. In that case, it requires linguists to be aware of a need to conduct in-depth analysis considering pragmatic mechanisms (Levinson, 1983; Pratama et al., 2017). Consequently, the realization of effective communication that is achieved *via* understanding and interpreting interlocutors’ utterances and responding properly requires the development of pragmatic competence involving the knowledge of implicatures. It is claimed that knowing linguistic forms and functions is not enough for effective communication. The language users need to use them appropriately to have socioculturally appropriate interaction.

### Comprehension of conversational implicatures and reading comprehension

Reading is one of the most crucial skills to be adept at succeeding in academic fields. Learners’ academic achievement is mainly related to their reading comprehension skills (Grabe, 1991; Bastug, 2014; Hijazi, 2018). It is considered an indispensable skill to comprehend what they read and to have better success in other subjects offered at a university level (Meniado, 2016). Although reading skill seems straightforward due to being provided with the required information, it consciously requires a serious process in constructing meaning from the text. In the process of reading, readers are required to comprehend the text by the help of decoding the writer’s words and constructing an approximate understanding of the writer’s intended message (Johnston and Kirby, 2006). To achieve success in reading skill, the reader is required to construct intended meaning, analyse and evaluate content for accuracy, make relevant connections with background knowledge and life experiences, and, most importantly, detect the implied meaning of the text. In this case, reading comprehension is a cognitively complex activity. It is like constructing a bridge between interlocutors.

Reading comprehension basically comprises understanding and interpretation of the written text. For that to be accomplished, various issues must be taken into account to be able to accurately understand what is read like, profoundly focusing on the intended meaning by considering what it actually means, and making connections between the written material and what is already known. Constructing the meaning of the written text is not straightforward; it requires reaching between and beyond lines to comprehend both explicitly stated and implied meaning. All those make the comprehension of written text a cognitively complex activity. The reading comprehension process not only necessitates decoding words, but it also makes use of focusing on the implied meaning, like the basic requirements of effective communication, which involve comprehending what is said and why it is said. Therefore, lacking comprehending the implied meaning of the written text will cause just viewing the words rather than comprehending the reading text. Keene and Zimmermann (1997) put emphasis on reading comprehension by saying that “teaching reading comprehension is mostly about teaching thinking.” Therefore, the mentioned considerations make reading comprehension a complex process requiring a reader to make a conscious effort to comprehend the intended meaning of the written text (i.e., understanding fully implied meaning by interpreting between and beyond the lines of the text). However, not being able to make a connection between what the author says and what he/she actually means can result in misinterpretation of the written text.

Thus, special emphasis should be placed on developing students’ conversational implicature to promote their understanding of both what is said and why it is said. So as to promote their reading comprehension abilities, conversational implicatures can be utterly beneficial. It is agreed on the fact that although EFL learners face hurdles in reading comprehension tests (Al Seyabi and Tuzlukova, 2015; Chen and Chen, 2015; Cho and Brutt-Griffler, 2015; Guimba and Alico, 2015; Hamra and Syatriana, 2015), the number of studies based on clarifying the impacts of pragmatic competences on students’ achievement remains very limited. It is essential to consider that language learners are required to be pragmatically qualified to be proficient in the target language since one of the most important components of language proficiency is undeniably the knowledge of pragmatics. Although there are various ways of developing L2 learners’ reading skills, the students generally face dilemmas to succeed in reading tests so that they may have poor achievement in reading comprehension. More importantly, focusing on grammatical and lexical aspects is not enough to promote students’ achievement; therefore, this shows that education on pragmatic competence has been neglected in the realm of EFL. Moreover, a recent study revealed that comprehending pragmatic meaning is more demanding than linguistic meaning (Liontou, 2018). Therefore, pragmatic inference is a crucial factor in text understanding. Although various issues may directly or indirectly affect students’ language performances like motivation, aptitude, age, gender, learners’ preferred learning styles, teachers’ teaching styles and so on, students’ pragmatic competence in L2 is pivotally required to take into account in facilitating language learning too.

According to our knowledge, the scarcity of existing literature on the relationship between comprehension of conversational implicatures and reading comprehension, this study is original in that it aims to fill a gap in the literature by investigating to what extent there is a statistical relationship between comprehension of conversational implicatures and comprehension of reading text. In this regard, the following research questions were formulated to guide the study:

1. Is there a significant correlation between students’ comprehension of conversational implicatures and their academic achievement in reading comprehension?
2. Does students’ comprehension of conversational implicatures predict their achievement in reading comprehension?
3. Are there any correlations between students’ scores on comprehension of conversational implicature types and their reading test scores in terms of reading subskills?

It has been hypothesized that students’ poor performance in the multiple-choice discourse completion test on the comprehension of conversational implicatures results in poor achievement in reading comprehension. Accordingly, poor achievement in comprehension of conversational implicatures and reading comprehension will have a pivotal impact on their academic achievement in other courses in their fields.

## Research methodology

### Research design

The current study employed a quantitative research approach due to the nature of the research objectives. Furthermore, in accordance with the research objectives, a correlational research design was used.

### Purpose of the study

The purpose of the study is to clarify, to what extent, comprehension of conversational implicatures is related to students’ reading comprehension achievement in a sample of 122 first-year university students. In this regard, comprehension of conversational implicatures has been mainly taken into account to investigate whether the participants’ scores in the comprehension of conversational implicatures are statistically correlated to their achievement in the reading comprehension test. In the light of the results, if there is a significant correlation between them, it emerges to integrate conversational implicatures into the EFL context.

### Participants

Data were collected from first-year students at one of the private universities in northern Cyprus with different bachelor’s degrees. The participants of the study were 122 undergraduates from the Faculties of Engineering, Law, Communication, Faculty of Fine Art, Design and Architecture, School of Health Sciences, Faculty of Arts and Sciences, and Faculty of Pharmacy. Cluster random sampling was utilized in the selection of participants, and they all volunteered to participate in the current study. They were all in their first year at university, and all of the participants had passed the English proficiency exam of the university, designed following the criteria of the Common European Framework of Reference for Languages (CEFRL) at the B2 level. The participants were made up of 58 males and 64 females ranging ages between 18 and 22 from Africa, Jordan, Iran, Iraq, Turkey, Syria and Palestine. Of the total population, 43.4% of participants (53 participants) were from Asia, 32.8% participants (40 participants) from Africa and 23.8% participants (29 participants) from Europe. While conducting the study, it was essential to ensure that all ethical policies were adhered to. Therefore, the ethical approvals of the study were obtained from the Ethic Committee of the university, and informed consent was obtained from all participants before the study began.

### Instruments and procedures

This study was conducted on the basis of two data collection instruments: MCDCT and the Reading Comprehension Test. To achieve the purpose of the study, 122 participants were asked to answer the Reading Comprehension test after being administered the MCDCT. Both data collection instruments were designed in the multiple-choice format as the marking procedure is required to be quite objective to provide reliability.

#### The multiple-choice discourse completion test

The first instrument for gathering information about the participants’ comprehension of conversational implicatures, a multiple-choice discourse completion test (MCDCT) was applied to the selected sample. Choosing this specific type of instrument provided practicality in terms of administering and scoring the gathered data. Besides the concern of practicality, a multiple-choice discourse completion test is more appropriate for collecting and analysing data from large groups. Before the adoption of the instrument, permission was granted from the owners of the test, Çetinavcı and Öztürk (2017). The test aims to investigate specifically the interpretation of implicatures (implied meanings) in English. It contains thirty-three scenarios which include eight different types of conversational implicatures. Each scenario comprised of a description of the situation and a dialogue in which a specific implied meaning was used (see Table 2). The utterance containing the implied meaning is written in bold and is followed by four options. Each option in each scenario represents a different interpretation of the target utterance. To determine the test takers’ comprehension of conversational implicatures, each correct answer is worth one point, and the other three incorrect options are assigned 0 points. This data collection instrument is to be used to investigate specifically the interpretation of implicatures (implied meanings) in English. It requires the test takers to read a written description of a situation in the target language and select what would be the most appropriate option to say in the given situation. Therefore, the students are required to choose the most appropriate response from the given four options for each scenario.

**Table 2:** Sample item from MCDCT.

Item # 1 (Conversational implicature type: indirect criticism)
Two teachers are talking about a student’s term paper
Mr. Ranger: Have you finished with Mark’s term paper yet?
Mr. Smith: Yes, I have. I read it last night.
Mr. Ranger: What did you think of it?
Mr. Smith: **Well, I thought it was well-typed.**
*How did Mr. Smith like Mark’s term paper?*
a. He liked it. He thought it was good.
b. He thought it was important that the paper was well-typed.
c. He really did not read it well enough to know.
d. He did not like it.

According to the data gathered by the analysis of SPSS 22, the Cronbach Alpha’s Reliability Coefficient was found to be as “0.756′, which can be accepted as highly reliable to use. However, the reliability of the test was checked again and the reliability of the MCDCT was piloted rigorously with a group of 132 different comparison groups through test–retest correlation and the results revealed that students’ pre-test scores and post-test scores were significantly correlated [*r*(130) = 0.756, *p* < 0.01]. It shows that the obtained scores were consistent across time and can be still accepted as highly reliable to use.

The following list, which has been mentioned in Section 2.3, includes the devices for communicating implied meaning covered in this present study:

X1 Pope Questions
X2 Indirect Criticism
X3 Topic Change
X4 Indirect Advice
X5 Verbal Irony
X6 Indirect Refusal
X7 Disclosure
X8 Indirect Requests (Requestive Hints)

Understanding implied speaker intention requires the interlocutor to use linguistic knowledge, contextual clues, and the assumption of relevance (Grice, 1975; Levinson, 1983; Sperber and Wilson, 1995; Thomas, 1995; Taguchi, 2005). As shown in the example above, “Understated Negative Evaluation” is used by saying, “Well, I thought it was well-typed. To understand the implied meaning, which is the sarcasm in this conversation rather than an appraisal expression from Mr. Smith for Mark, comprehension of conversational implicature may be influenced by lack of correct understanding of the vocabulary or the grammatical constructions of the items in the test (Carrell, 1984; Kasper, 1984; Takahashi and Roitblat, 1994; Garcia, 2004; Roever, 2005; Taguchi, 2005, 2007; Holtgraves, 2007; Eslami and Eslami-Rasekh, 2008; Abdelhafez, 2016). Thus, interlocutors need to know the vocabulary and grammar of the items in the conversational implicatures to comprehend the implied meaning.

#### The reading comprehension test

The Reading Comprehension Test used in the study is one of the IELTS (International English Language Testing System) Reading Test, which is entitled “A neuroscientist reveals how to think differently (IELTS Mentor, n.d.).” ‘International English Language Testing System’ (IELTS) reading comprehension test was conducted in order to gather data on the achievement of test takers’ reading comprehension. In this respect, this study aims to analyze to what extent the test takers are able to select the best option for each multiple-choice formatted reading comprehension question.

The IELTS Reading Test (A neuroscientist reveals how to think differently) consists of fourteen questions based on the passage (see Table 3) and six questions have been added to the test (see Table 4). The reading test comprises six parts which aim to determine the students’ abilities on the basis of the following points:

**Table 3:** Sample item from the reading comprehension test.

Item # 1 (Reading subskill type: to scan a text to find specific information)
1. Neuroeconomics is a field of study which seeks to
A. cause a change in how scientists understand brain chemistry.
B. understand how good decisions are made in the brain.
C. understand how the brain is linked to achievement in competitive fields.
D. trace the specific firing patterns of neurons in different areas of the brain.

**Table 4:** Sample item from the reading comprehension test.

Item # 19 (Reading subskill type: to make inferences to identify irrelevant sentences)
19. Science is systematic because of the attention it gives to organizing knowledge and making it readily accessible to all who wish to build on its foundation. If the results support the hypothesis, the scientist may use them to generate related hypotheses. In this way, science is both a personal and a social endeavour. In other words, it is beneficial both to the individual and to society at large. Therefore, science contributes a great deal to the improvement and the quality of human life.
A. Science is systematic because of the attention it gives to organizing knowledge and making it readily accessible to all who wish to build on its foundation.
B. If the results support the hypothesis, the scientist may use them to generate a related hypothesis.
C. In this way science is both a personal and a social endeavour.
D. In other words, it is beneficial both to the individual and to society at large.

Y1: Reading subskill to scan a text to find specific information (Item 1, Item 2, Item 3, Item 4, Item 5).

Y2: Reading Subskill to identify ideas and opinions of the writer (Item 6, Item 7, Item 8, Item 9, Item 10, Item 11).

Y3: Reading Subskill to comprehend particular points or overall idea of the passage (Item 12, Item 13, Item 14).

Y4: Reading Subskill to skim a paragraph to identify a topic (Item 15, Item 16).

Y5: Reading Subskill Ability to skim a paragraph to identify a topic sentence (Item 17, Item 18).

Y6: Reading Subskill to make inferences to identify irrelevant sentences (Item 19, Item 20).

In this regard, the second instrument is comprised of a twenty-multiple-choice formatted reading test. The participants need to read and answer the questions in a forty-minute duration. To ensure the reliability and validity of the reading test, the researcher conducted an in-depth analysis.

The reliability of the reading test (the IELTS reading passage) was checked on a group of 88 students through test–retest correlation. One of the most common ways of assessing reliability is measuring ‘stability or test–retest’. To measure the stability of a test, the participants were delivered the same test twice with a probable interval of three weeks and their scores were calculated to determine the correlation between these two tests in which reliability was reflected. To determine the participants’ achievement in reading comprehension, each correct answer is worth 1 point, and the other three incorrect options are assigned a ‘0’ point. The results revealed that students’ pre-test scores and post-test scores were significantly correlated [*r*(86) = 0.931, *p* < 0.01]. This correlation value implies that the reading test is reliable due to its consistency. It means that test-takers perform almost the same at all times when it is delivered. Moreover, in terms of the practicality of the instrument, during the process of the pilot study, the participants are asked to make comments about the wording, timing and their understanding of the items.

With regard to the validity of the test, this test is regarded as valid due to measuring what is expected to measure in an efficient way. In relation to improving the validity of the reading test, the researcher paid close attention to the following issues: main goals and objectives were clearly defined by using precise language, the goals and objectives of the study were analysed to determine to what extent they match, troublesome wording or other difficulties were edited for not destroying the validity of the test, and also the test was compared with other reading comprehension tests. In this sense, the validity of the instrument is regarded as being more important than its reliability since a reliable instrument may lack the validity. In terms of achieving the content validity of the reading test, an extensive search of the literature was held by the researcher. Related previous instruments and past research findings were inspected. In what follows, some of these studies were reviewed. In the study of Xiao and Lin (2015), the content validity of a number of reading comprehension tests was analysed to determine whether they were appropriate for the New Curriculum Standard and Testing Syllabus command. The researchers analysed the aspects of length, reading speed, the proportion of new words, readability, genre and reading skills. The study suggested redesigning the order of reading texts more reasonably. Another study was carried out by Hu and Wei (2016) on the content validity of reading comprehension tests. They suggested that English teachers should increase reading exercises of practical writing and put more emphasis on passage structures and attitude. Moreover, Qi (2019) conducted a non-empirical investigation to analyse the content validity of the reading part of TEM4 (the Test for English Majors). The findings proved that the content validity of the reading comprehension part is crucial to the quality of the test for English majors. The researcher asserted that the test syllabus and its specifications in terms of instructions, text length and content should be considered.

In addition to the content validity, face validity was inspected in accordance with comparing the test with what is supposed to be assessed. More importantly, three professors who are experts in this area have approved the validity of the test. They have all agreed that the test they inspected is valid regarding face validity, content validity and validity of generalizability. Therefore, regarding experts’ comments on the reading test, this reading test can be used to reflect logical evidence to support the interpretation of students’ reading comprehension scores.

### Results

This section encompasses the findings and discussions related to the research questions. The results of the study have been analysed to investigate whether comprehension of conversational implicatures is related to students’ reading comprehension achievement. The results of the study have been shown into three discrete sections. The first section includes an analysis based on whether a correlation exists between students’ comprehension of conversational implicatures and their achievement in reading comprehension. The second section is derived from an analysis based on to what extent participants’ comprehension of conversational implicatures predicts their achievement in reading comprehension and the final section is about whether there are any correlations between students’ scores on comprehension of conversational implicature types and their reading test scores in terms of reading subskills. All statistical analysis was carried out by applying the Statistical Package for Social Sciences program. The results of the study are delineated in the following data:

#### The correlation between participants’ MCDCT and IELTS reading test scores

The first research question of the study aims to determine whether a correlation exists between students’ comprehension of conversational implicatures and their achievement in reading comprehension. As depicted in Table 5, a Pearson Correlation Analysis has been conducted to analyze the relationship between the students’ achievement in the reading comprehension test and conversational implicatures comprehension.

**Table 5:** The correlation between participants’ MCDCT and IELTS reading test scores.

		Scores of MCDCT	Scores of IELTS reading test
Scores of MCDCT	Pearson correlation	1	0.896^**^
	Sig. (2-tailed)		0.000
	N	122	122
Scores of the IELTS reading test	Pearson correlation	0.896^**^	1
	Sig. (2-tailed)	0.000	
	N	122	122

** Correlation is significant at the 0.01 level (2-tailed).

Based on the results of the study, comprehension of conversational implicatures correlates positively with reading comprehension, *r* = 0.896, *n* = 122, *p* = 0.000. The correlation of achievement in the reading test with itself is (*r* = 1) and the number of students who take the test is *n* = 122. The correlation between students’ achievement in the reading test and their performance in the comprehension of conversational implicatures is (*r* = 0.896), based on *n* = 122. Thus, the results revealed that the comprehension of conversational implicatures and the achievement in the reading test have a statistically significant linear relationship (*p* = 0.000). The direction of the relationship is positive as comprehension of conversational implicatures and achievement in the reading test are positively correlated. In this sense, these variables tend to increase together. This finding suggests that a learner’s comprehension of conversational implicatures is crucial in predicting one’s reading comprehension achievement. Therefore, it is utterly crucial to develop language learners’ pragmatic competence regarding conversational implicatures to help them succeed in the reading comprehension tests effectively.

#### Predicting L2 learner’s achievement in reading comprehension *via* their conversational implicature comprehension

The second section is derived from an analysis based on to what extent the participants’ comprehension of conversational implicatures predicts their achievement in reading comprehension.


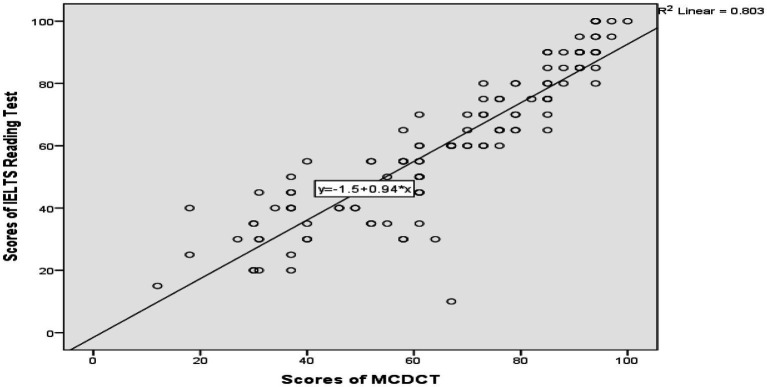


A simple linear regression analysis has been conducted to evaluate whether the students’ reading comprehension achievement is affected by their comprehension of conversational implicatures. The scatterplot for the two variables indicates that the two variables are linearly related as the students’ success increases in relation to the increase in the students’ comprehension of conversational implicatures. The regression equation for predicting the overall comprehension of conversational implicatures of the students is.

*Predicted Overall IELTS Reading Test Score of Students* = 1.5 + 0.94 *(Comprehension of Conversational Implicatures of Students).*

With the 95% confidence interval for the slope, students’ overall achievement in the IELTS reading test results is significantly related to their comprehension of pragmatic competencies based on conversational implicatures. As hypothesized, the students who have high scores from MCDCT significantly outperform in the IELTS reading test. In this sense, the students who have poor achievement scores from MCDCT are not able to succeed in the IELTS reading test as depicted in the study. The correlation between the students’ achievement in the reading test and their comprehension of conversational implicatures is 0.89. Thus, the participants’ comprehension of conversational implicatures affects their reading comprehension with an 89% contribution.

#### Correlations between reading test subskills and conversational implicature types

The third research question aims to find out if any correlations exist between the two sets which are the participants’ comprehension according to types of conversational implicature and their reading comprehension considering the reading subskills. The Canonical correlation analysis has been conducted to determine the relationships between the two different sets. Table 6 depicts statistical data about to what extent each variable affects the whole relationship.

**Table 6:** Correlation between reading test subskills and conversational implicature types.

	Reading subskill to scan a text to find specific information	Reading subskill to identify ideas and opinions of the writer	Reading subskill to comprehend particular points or overall idea of the passage	Reading subskill to skim a paragraph to identify a topic	Reading subskill to skim a paragraph to identify a topic sentence	Reading subskill to make inferences to identify irrelevant sentences
Pope question	0.249^**^	0.206^*^	0.298^**^	0.203^*^	0.188^*^	0.342^**^
Indirect request	0.342^**^	0.372^**^	0.356^**^	0.307^**^	0.231^*^	0.419^**^
Topic change	0.521^**^	0.613^**^	0.499^**^	0.558^**^	0.202^*^	0.441^**^
Indirect advice	0.285^**^	0.329^**^	0.379^**^	0.313^**^	0.292^**^	0.543^**^
Verbal irony	0.440^**^	0.433^**^	0.407^**^	0.424^**^	0.191^*^	0.434^**^
Indirect refusal	0.397^**^	0.330^**^	0.456^**^	0.476^**^	0.372^**^	0.467^**^
Disclosure	0.379^**^	0.305^**^	0.240^**^	0.247^**^	0.291^**^	0.316^**^
Indirect request	0.348^**^	0.329^**^	0.266^**^	0.212^*^	0.241^**^	0.250^**^

** Correlation is significant at the 0.01 level (2-tailed).

* Correlation is significant at the 0.05 level (2-tailed).

As can be seen in Table 6, there are only three moderate positive correlations. The highest moderate positive correlation is between reading subskill to identify ideas and opinions of the writer and topic change [*r*(122) = 0.613, *p* < 0.001]. It is followed by a moderate positive correlation between reading subskill to skim a paragraph to identify a topic and topic change [*r*(122) = 0.558, *p* < 0.001]. Similarly, the third moderate positive correlation takes place between reading subskill to make inference to identify irrelevant sentence and indirect advice [*r*(122) = 0.543, *p* < 0.001]. The other correlations can be considered low positive correlation due to the values of correlation coefficient. They differ in range between 0.30 and 0.50.

Table 7 shows correlation results within the comprehension of conversational implicature types. All the conversational implicature types have significant relationships with other conversational implicature types, but the highest significant correlation coefficient is 0.518 and the lowest significant correlation coefficient is 0.078. The study reveals that there are low positive correlations among most of the conversational implicature types. In addition, there is only one moderate positive correlation that exists between verbal irony and topic change. It is crucial to point out that although most of the conversational implicature types are significantly correlated within themselves, correlations are neither high nor very high.

**Table 7:** Correlation results within comprehension of conversational implicature types.

	X1	X2	X3	X4	X5	X6	X7	X8
X1	1							
X2	0.467^**^	1						
X3	0.219^*^	0.386^**^	1					
X4	0.431^**^	0.346^**^	0.442^**^	1				
X5	0.137	0.162	0.518^**^	0.299^**^	1			
X6	0.244^**^	0.295^**^	0.355^**^	0.379^**^	0.450^**^	1		
X7	0.126	0.268^**^	0.172	0.180^*^	0.253^**^	0.240^**^	1	
X8	0.186^*^	0.277^**^	0.394^**^	0.242^**^	0.180^*^	0.363^**^	0.078	1

** Correlation is significant at the 0.01 level (2-tailed).

* Correlation is significant at the 0.05 level (2-tailed).

Table 8 shows correlation results within reading test subskills. The results indicate that all of the subskills are significantly correlated within themselves. The range of correlation coefficient scores is from 0.179 to 0.749.

**Table 8:** Correlation results within reading test subskills.

	Y1	Y2	Y3	Y4	Y5	Y6
Y1	1					
Y2	0.749^**^	1				
Y3	0.545^**^	0.614^**^	1			
Y4	0.551^**^	0.439^**^	0.568^**^	1		
Y5	0.311^**^	0.179^*^	0.237^**^	0.251^**^	1	
Y6	0.384^**^	0.231^*^	0.475^**^	0.542^**^	0.407^**^	1

** Correlation is significant at the 0.01 level (2-tailed).

* Correlation is significant at the 0.05 level (2-tailed).

This shows that the range of correlations is different from low positive correlation to high positive correlation. There is only one high positive correlation that is between the reading subskill to identify ideas and opinions of the writer (Y2) and the reading subskill to scan a text to find specific information [Y1; *r*(122) = 0.749, *p* < 0.001]. In addition to this, there are five moderate positive correlations that are listed below:

- Reading subskill to scan a text to find specific information (Y1) and reading subskill to comprehend particular points or overall idea of the passage (Y3)
- Reading subskill to scan a text to find specific information (Y1) and reading subskill to skim a paragraph to identify a topic (Y4)
- Reading subskill to identify ideas and opinions of the writer (Y2) and reading subskill to comprehend particular points or overall idea of the passage (Y3)
- Reading subskill to skim a paragraph to identify a topic (Y4) and reading subskill to comprehend particular points or overall idea of the passage (Y3)
- Reading subskill to skim a paragraph to identify a topic (Y4) and reading subskill to make inferences to identify irrelevant sentences (Y6)

These correlation results indicate that most of the reading subskills have positive relationships within themselves. In addition, being competent in one of these subskills will contribute to the achievement of the other subskill.

In Table 9, canonical correlation results provide correlation coefficients for canonical variables, Wilk’s values, and the significance of the canonical variables. There are six canonical variables, and their correlation coefficients differ in the range from 0.081 to 0.833. Except for the first canonical variable, the remaining canonical variables are not found as significant. The first canonical variable is significant at the 0.01 level (*r*_uv1_ = 0_._833, Wilk’s Lambda = 0.201, *p* < 0.01). This shows that the first canonical variable is meaningful for further exploration.

**Table 9:** Canonical correlation statistics.

Canonical correlations							
	Correlation	Eigenvalue	Wilks statistics	*F*	Num D.F	Denom D.F.	Sig.
1	0.833	2.268	0.201	4.300	48.000	535.468	0.000
2	0.411	0.203	0.657	1.382	35.000	460.951	0.075
3	0.321	0.115	0.791	1.118	24.000	384.954	0.320
4	0.300	0.099	0.881	0.957	15.000	306.824	0.501
5	0.157	0.025	0.969	0.447	8.000	224.000	0.892
6	0.081	0.007	0.993	0.251	3.000	113.000	0.860

In Tables 10, 11, standardized canonical coefficients for canonical variables are provided. Equations for the canonical variable of Set-1 and Set-2 are shown below.

**Table 10:** Canonical coefficients for Set 1.

Set 1 standardized canocial correlation coefficients						
Variables	1	2	3	4	5	6
Pope questions	−0.038	−0.123	0.328	−0.056	0.827	0.360
Indirect criticism	−0.183	−0.169	−0.427	0.005	−0.053	0.103
Topic change	−0.421	1.089	−0.094	0.167	−0.235	0.200
Indirect advice	−0.199	−0.744	−0.649	0.280	−0.467	−0.092
Verballrony	−0.195	−0.141	−0.298	−0.262	0.813	−0.508
Indirect refusal	−0.216	0.001	0.897	0.766	−0.222	0.017
Disclosure	−0.218	−0.129	0.339	−0.608	−0.325	−0.512
Indirect request	−0.045	−0.221	0.140	−0.753	−0.118	0.585

**Table 11:** Canonical coefficients for set 2.

Set 2 standardized canonical correlation coefficients						
Variable	1	2	3	4	5	6
Sub skill to scan a text to find specific information	0.003	−0.007	1.092	−0.483	1.159	−0.216
Sub skill to identify ideas and opinions of the writer	−0.510	0.464	−0.908	−0.739	−0.991	0.031
Sub skill to understand particular points or over all idea of the passage	−0.029	−0.206	0.324	0.587	0.266	1.250
Sub skill to skima paragraph to identify a topic	−0.182	0.812	−0.133	0.877	−0.143	−0.640
Sub skill to skima paragraph to identify a topic sentence	−0.095	−0.214	0.655	0.140	−0.857	−0.096
Sub skill to make inference to identify irrelevent sentence	−0.532	−0.863	−0.690	−0.281	0.247	−0.314

*Comprehension of Conversational Implicatures (U1)* **=** −0.038X1 − 0.183X2 − 0.421X3 − 0.199X4 −0.195X5 − 0.216X6 −0.218X7 − 0.045X8

*Reading Comprehension (V1)* **=** 0.003Y1 − 0.510Y2 −0.029Y3 − 0.182Y4 − 0.095Y5 − 0.532Y6

When the first canonical variable (U1) of set-1 is analysed, it is depicted that topic change (X3), indirect refusal (X6) and disclosure (X7) provide the most contribution (0.855) to comprehension of conversational implicatures. It means that the three conversational implicature types provide the most contribution to reading comprehension, and they influence their reading achievement with 85.5% contribution. Results show that 1-point change in X3, X6 or X7 will lead to 0.421, 0.216 and 0.218-point change in U1. On the other hand, when the canonical variable (V1) of set-2 is analysed, the ability to make inferences to identify irrelevant sentences (Y6) and the ability to identify ideas and opinions of the writer (Y2) provide the most contribution (1.042) to the participant’s reading comprehension (V1). These results also show that 1-point change in Y6 and Y2 will lead to 0.532 and 0.510-point change in V1.

Table 12 shows redundancy analysis results for canonical variables. Redundancy analysis results show the proportion of variance explained by its own set and the opposite set of a variable. Redundancy analysis results show 36.9% of the first set of the canonical variable (U1) and 25.6% of the first set of canonical variables (U1) that is explained by the opposite canonical variable (V1). When the redundancy analysis results for the second set of canonical variables (V1) is analysed, it shows that 50.3% of the second set of canonical variables (V1) is explained by itself and shows that 34.9% of the second set of canonical variables (U1) is explained by the opposite canonical variable (X).

**Table 12:** Redundancy analysis results.

Propotion of variance explained				
Canonical variable	Set 1 by self	Set 1 by set 2	Set 2 by self	Set 2 by set 1
1	0.369	0.256	0.503	0.349
2	0.089	0.015	0.125	0.021
3	0.081	0.008	0.086	0.009
4	0.080	0.007	0.107	0.010
5	0.075	0.002	0.088	0.002
6	0.128	0.001	0.091	0.001

## Discussion and educational implicatures

This study offers several implications for the development of reading comprehension which is positively related to the development of L2 implicature comprehension. The first notable result relates to the relationship between comprehension of conversational implicatures and reading comprehension. This study reveals that comprehending reading text is much more than decoding or sounding out words. Making a connection between the letters, understanding their combinations to form the words, or figuring out the main focus of the group of sentences are not all it is needed to be able to succeed in reading comprehension tests. Instead, it requires us to find a relationship between what is said and why it is said. Therefore, reading comprehension is a highly complicated activity that requires active use of the mind to reason and understand the author’s intended message with interpretation, analysis, evaluation, and synthesis of its content. In this case, achieving success in reading comprehension is similar to comprehending conversational implicatures in which it is needed to understand the relationship between what you say and what you actually mean.

The first research question of the study is in support of this point, showing that comprehension of conversational implicatures and achievement in the reading test has a statistically significant linear relationship (*p* = 0.000). Although a correlational study is a non-experimental study in which two numerical variables are measured and assessed to determine their statistical relationships with little or no effort to manipulate, educational implications based on this point is that it is consequential to enrich the students’ abilities in comprehending conversational implicatures to promote their achievement in reading comprehension. It is important to take into account that although this correlational study does not determine which quantitative variable affects the other, one of the factors in creating dilemma in reading comprehension could arise due to the weaknesses in comprehending conversational implicatures. This study proves that students’ success in one variable triggers the other variable. Thus, this issue sheds light on the EFL context to integrate various language activities to develop their ability to comprehend conversational implicatures and promote their reading comprehension achievement. Bearing in mind that although students’ achievement in reading comprehension can be affected by their proficiency level of English, their ability to comprehend conversational implicatures in English as an L2 can be associated with their achievement in reading comprehension.

The second research question is based on to what extent participants’ comprehension of conversational implicatures predicts their achievement in reading comprehension. The scatterplot for the two numerical variables, participants’ scores in the reading comprehension test and their scores in the conversational implicature comprehension, proved that they are linearly related. With the 95% confidence interval for the slope, participants’ overall achievement in the reading comprehension test is significantly related to their comprehension of pragmatic competence based on conversational implicatures. On the basis of this analysis, it could be predicted that students’ overall reading comprehension achievement can be determined *via* comprehension of conversational implicatures. It could be hypothesized that having a high score in the comprehension of conversational implicature may lead to a high score in reading comprehension. However, the following analysis sought to explain to what extent types of conversational implicatures best explain achievement in reading comprehension among the selected population.

In the analysis of the third research question, the Canonical Correlation has been carried out to show whether there are correlations between two sets. The first set is made up of comprehension of conversational implicature types: pope questions (X1), indirect criticism (X2), topic change (X3), indirect advice (X4), verbal irony (X5), indirect refusal (X6), disclosure (X7) and indirect request (X8). The variables in the second set are subskill to scan a text to find specific information (Y1), subskill to identify ideas and opinions of the writer (Y2), subskill to comprehend particular points or overall idea of the passage (Y3), subskill to skim a paragraph to identify a topic (Y4), subskill to skim a paragraph to identify a topic sentence (Y5) and subskill to make inference to identify irrelevant sentences (Y6). Both groups are predictor variables in terms of depicting to what extent each variable affects the whole relationship. The study shows that there is a significant correlation at the 0.01 level between the Reading test and the MCDCT test according to the canonical correlation results (ruv1 = 0.833, *p* < 0.01). According to the Redundancy analysis results, the MCDCT test explains the 25.6% variance of the Reading test and the Reading test explains the 36.9% variance of the MCDCT test. Furthermore, 50.3% variance in the Reading test is explained by itself, and 36.9% variance in the MCDCT test is explained by itself. The canonical correlation analysis reveals that Y2 (subskill to identify ideas and opinions of the writer) and Y6 (subskill to make inferences to identify irrelevant sentences) variables provide the most contribution to the achievement in reading comprehension. Furthermore, X3, X6 and X7 are the variables that provide the most contribution to the students’ comprehension of conversational implicatures due to the results of the MCDCT test. In this sense, this study stipulates that L2 students’ achievement in reading comprehension is related to their comprehension of conversational implicatures, and the development of the three conversational implicature types which are topic change (X3), indirect refusal (X6), and disclosure (X7) provides more contribution to students’ success in reading comprehension.

## Conclusion

In conclusion, growing emphasis on the importance of English and the necessity to gain communicative competence in the target language has resulted in the rapid change in teaching English as a foreign language globally. Various studies have been conducted to promote L2 learners’ competence in the target language in terms of helping them maintain effective communication by understanding what is said and why it is said. As it is, the last decade has seen significant advancements in L2 comprehension of implicature studies. This study draws attention to the pedagogical implications of training language learners concerning conversational implicatures to promote their achievement in reading comprehension. Reading skill is regarded as the cornerstone of the learning process (Ramrathan and Mzimelaj, 2016). It is consequential to develop the students’ abilities to be able to comprehend written texts. This study proves that not only the students’ target proficiency level is the prominent factor, but also their comprehension of conversational implicatures in the target language has a significant association with reading comprehension. Thus, the weaknesses in comprehending conversational implicatures can result in potential dilemmas in the achievement of reading comprehension. As Roe (2014) states, reading comprehension is a process that requires us to make meaning of what we read. Although there are various studies on the importance of conversational implicatures, there is still a huge gap in the literature about to what extent the EFL learners’ abilities in terms of comprehending conversational implicatures are related to their achievement in reading comprehension. In this sense, this study has been conducted to investigate whether there is a relationship between L2 students’ comprehension of conversational implicatures and their achievement in reading tests. The results of the study revealed that comprehension of conversational implicatures of first-year university students is positively related to their achievement in reading comprehension. The current study proves that the participants’ achievement in reading comprehension and their comprehension of conversational implicatures increase together. Thus, this finding supports that in the goal of facilitating second language learners’ achievement in reading comprehension, this study creates utterly crucial awareness in terms of the importance of integrating pragmatics into language learning.

In the light of the findings of the third research question, it has been depicted that among the eight implicature types, “Topic Change,” “Indirect Refusal,” and “Disclosure” are more related (0.855) to reading comprehension. Since these three implicature types contribute the most to the participants’ comprehension of conversational implicatures, they contribute more to students’ achievement in reading comprehension tests. It was depicted that “Topic Change” (0.421) provides the most contribution to students’ achievement in a reading comprehension test among all conversational implicature types. It is a type of implied meaning, and it happens when an interlocutor feels that a current line of conversation is inappropriate. Therefore, the interlocutor prefers to leap into another topic. In the reading comprehension process, the students need to correctly comprehend what is happening in the text by figuring out relevant and irrelevant answers according to the given set of reading comprehension questions. Without comprehension, it is difficult to understand what the text says and why it says. Likewise, comprehending reading texts involves reading, understanding, and answering a set of questions. The reading passage is made up of a number of paragraphs about any topic, and the students need to understand the main ideas contained in a text to be able to look for specific information in the correct paragraph of the reading text. Moreover, the results show that among the eight implicature types which provide the most contribution to students’ achievement in a reading comprehension test, Topic Change (0.421) is followed by Indirect Refusal (0.216) and Disclosure (0.218). In this sense, they influence the student’ reading comprehension achievement with an 85.5% contribution. Indirect Refusal is one of the types of conversational implicatures in the light of the pertinent literature. Indirect Refusal happens when the recipient disapproves of the interlocutor’s idea by providing reasons or explanations. This ability helps individuals to figure out the connection between the situation and why the interlocutor cannot fulfil the speaker’s demand. Accordingly, students’ reading comprehension achievement can be promoted by connecting all the links. Disclosure is another type of conversational implicature that contributes to the students’ reading comprehension achievement with 21.8%. Interlocutors sometimes use indirect replies to avoid disclosing embarrassing information (Taguchi, 2002, p. 157). This implicature type encourages students to go beyond understanding information given in the reading text by understanding the implied level meaning.

Considering the fact as mentioned earlier, conversational implicatures are vital to integrate them into the EFL context to promote students’ achievement in reading comprehension. This present study suggests a direction for future teaching and learning in promoting students’ achievement in reading comprehension. Developing L2 learners’ conversational implicatures seems conducive to cultivating their reading comprehension. Depending on the fruitful evidence that has been provided above, integrating pragmatics into the arena of foreign language learning is seriously required to be taken into account by language teachers, textbook writers, and curriculum designers. In this sense, researchers are required to develop effective methods to cultivate students’ success in reading tests, performances in language learning, and success in their other courses. Thus, this study sheds light on the enlightenment in the language teaching and learning era.

## Data availability statement

The original contributions presented in the study are included in the article/supplementary material, further inquiries can be directed to the corresponding author.

## Ethics statement

Ethical review and approval was not required for the study on human participants in accordance with the local legislation and institutional requirements. Written informed consent from the patients/participants or patients/participants legal guardian/next of kin was not required to participate in this study in accordance with the national legislation and the institutional requirements.

## Author contributions

SÇ and ÖD contributed equally to the manuscript generation, writing process, and approved the submitted version.

## Conflict of interest

The authors declare that the research was conducted in the absence of any commercial or financial relationships that could be construed as a potential conflict of interest.

## Publisher’s note

All claims expressed in this article are solely those of the authors and do not necessarily represent those of their affiliated organizations, or those of the publisher, the editors and the reviewers. Any product that may be evaluated in this article, or claim that may be made by its manufacturer, is not guaranteed or endorsed by the publisher.
